# Prevalence of human Salmonellosis in Ethiopia: a systematic review and meta-analysis

**DOI:** 10.1186/1471-2334-14-88

**Published:** 2014-02-19

**Authors:** Getachew Tadesse

**Affiliations:** 1 Department of Biomedical Sciences, College of Veterinary Medicine and Agriculture, Addis Ababa University, P.O. Box 34, Debre Zeit, Ethiopia

**Keywords:** Ethiopia, Human, Prevalence, *Salmonella*, Serogroups, Serotypes

## Abstract

**Background:**

Human Salmonellosis is one of the major diseases in Ethiopia and several factors including under and mal-nutrition and HIV-AIDS may substantially contribute to its occurrence. Despite its importance, surveillance and monitoring systems are not in place and a comprehensive picture of its epidemiology is not available. The objectives of this study were to systematically review and estimate the prevalence of the disease and identify the dominant serogroups and serotypes in Ethiopia.

**Methods:**

Published studies on Salmonellosis in Ethiopia were electronically and manually searched. Eligible studies were selected by using inclusion and exclusion criteria. Generic, methodological and statistical information were extracted from the eligible studies. The extracted data included sample sizes, the numbers of *Salmonella* positive samples, serogroups and serotypes. The variations in prevalence estimates attributable to heterogeneities were assessed and pooled prevalence was estimated by the random effects model.

**Results:**

Twenty studies carried out between 1974 and 2012 were eligible. The pooled prevalence estimates of *Salmonella* in stool samples of diarrheic children, diarrheic adults and carriers were 8.72%, 5.68%, and 1.08% respectively. Invasive infections in children (5.71%) and adults (0.76%) were significantly different (p < 0.001). Non-typhi isolates accounted for 57.9% of the isolates from patients. Serogroup D occurred more frequently than serogroups C and B. *S*. Concord, *S*. Typhi, *S.* Typhimurium and *S*. Paratyphi were dominant and accounted for 82.1% of the serotypes isolated from patients.

**Conclusion:**

The prevalence of Salmonellosis is considerable and most infections are due to four serotypes. The results imply the need for a policy to promote public hygiene and regularly screen individuals in contact with food items for public consumption.

## Background

Salmonellosis is one of the major zoonotic diseases all over the world with annual estimates of 22 million cases and 200,000 deaths due to typhoid fever [1] and 93.8 million cases of gastroenteritis and 155 000 deaths due to non-typhoidal Salmonellae (NTS) [2]. In resource-poor settings of Africa, enteric fever is a public health concern [3] with an incidence of 10-100/100,000 cases per year [1]. Of the NTS, *S.* Typhimurium and *S.* Enteritidis are common [4,5], and in 2002 each accounted for approximately 25% of the human isolates [6].

Children, the elderly and immunocompromised individuals are the high risk groups and case fatality rates of 38% in children [7] and 47% in adults [8] were recorded. Whilst typhoid fever shows little association with immunocompromise, NTS infections in HIV positive adults are associated with severe invasive diseases [9]. Bacteremia and focal infections are more likely to occur in immunocompromised than in immunocompetent children and infectious arteritis is one of the complications in adults [10]. Higher occurrences of bacteremia were associated with Malaria [11] malnutrition [5,12] and recent antimicrobial use [13].

In Ethiopia, several factors including under and mal-nutrition, HIV-AIDS, the unhygienic living circumstances and the close relations between humans and animals may substantially contribute to the occurrence of Salmonellosis. Although surveillance and monitoring systems are not in place and its epidemiology is not described, qualitative and quantitative syntheses of previous studies could shed light on the occurrence of the disease and the major serotypes that frequently cause infections. The objectives of this study were to systematically review and estimate the prevalence of human Salmonellosis in Ethiopia by using meta-analytical methods. The outcomes of interests were the proportions of *Salmonella* isolated from diarrheal patients, febrile patients, apparently normal subjects and the dominant serogroups and serotypes.

## Methods

The guidelines of the MOOSE group (Meta-analysis of Observational Studies in Epidemiology) [14] and the PRISMA group (Preferred Reporting Items for Systematic Reviews and Meta-Analyses) [15] were followed in the reviewing. The PRISMA check list was used to ensure inclusion of relevant information (see Additional file 1).

### Eligibility criteria

A study was eligible for quantitative syntheses if (i) its objective was not serotype specific; (ii) it was cross sectional; (iii) the samples were taken from patients that sought medical attention for either reasons of gastroenteritis and/or fever in health care settings or from apparently healthy individuals; (iv) it provided the sample size; (v) it described the microbiological methods; (vi) it provided the numbers of isolates and (vii) it was published in English. Other studies with relevant information on serogroups, serotypes typhoidal and NTS isolates were included in the reviewing.

### Literature search strategies

Published studies were searched in Medline. Non-Medline indexed articles were searched in the lists of references of articles and by using Google scholars. Salmonell* and Ethiopia were the main MeSH terms in electronic searches. Additional searches were done by using the main MeSH terms with Boolean operators and other terms that included prevalence, incidence, antimicrobial resistance, human, animals, foods and Addis Ababa. The search covered articles published up to November 19, 2013.

### Selection of studies

Initially articles with titles and abstracts that were not relevant to the outcomes of interests were excluded. The full texts of all articles screened for eligibility but one were either downloaded or obtained from the corresponding authors (Dr. Byleyegne Molla, Dr. Endrias Zewdu and Mr. Bayeh Abera) or from the archives of the College of Health Sciences, School of Medicine, Addis Ababa University. Of the screened articles, duplicates and articles that did not meet the eligibility criteria were excluded.

### Data extraction

The first author, year of publication, year of study, location, setting, sample size, age group, types of samples, microbiological methods, numbers of *Salmonella* positive samples, serogroups and serotypes were extracted from the eligible studies. The proportions of typhoidal and non-typhoidal Salmonellae, serogroups and serotypes were calculated by using the numbers of isolates as denominators. A serotype was considered to be dominant if its occurrence was more than 5% of the total serotyped isolates.

### Data analysis

The data were stratified on the basis of feel of relative homogeneity [14] as hospital based and non-hospital based. The hospital based data were further grouped by sample type (stool and blood) and age group (adults and children). To produce conservative estimates, a zero reported for the number of *Salmonella* positive samples was imputed as 0.5 [16]. The study level prevalence estimates (p) and standard errors (se) were calculated by the following formulae: p = np/n and se. =√ p (1-p*)*/n: where np = the number of positive samples and n = the number of samples.

### Investigation of bias

The qualities of the microbiological methods of the studies were assessed to determine the extent to which bias is introduced and results in inferential error. The risks of bias across studies were assessed by funnel plots [17] and the Duval and Tweedie non-parametric 'trim and fill’ linear random model [18]. The funnel plots were used to visually examine outliers and the tendencies of studies with small sample sizes to show larger prevalence estimates. The Duval and Tweedie method was used to calculate the unbiased estimate.

### Heterogeneity analyses

The variations between studies were assessed by the Galbraith plot [19], the Cochran’s Q test and the Inverse variance index (I^2^) [20]. The Galbraith plot was used to graphically display the ratio of the prevalence estimates and the standard errors (p/se) against the reciprocals of the standard errors (1/se). Because of the reduced power of the Cochran’s Q test to test the between-study variation in small number of studies, the significance of the heterogeneities was tested at a 10% significance level [21]. The percentage of the variation attributable to heterogeneity was quantified by the Inverse variance index and values of 25%, 50% and 75% were considered as low, moderate and high heterogeneity respectively.

### Sensitivity tests

A sensitivity test was done to assess the effects of outliers [22]. The Duval and Tweedie non-parametric linear random effects model [18] and single study omitted influence analyses were done to assess the sensitivities of the pooled prevalence estimates of *Salmonella* in diarrheal patients and apparently healthy subjects. A study was considered to be influential if the pooled estimate without it was not within the 95% confidence interval of the overall pooled estimate.

### Quantitative data syntheses

To normalize the distribution of the data and incorporate the influence of sample size on the outcomes, the study level estimates were transformed to logit event estimates [23,24]. The logit event estimates and the variances were calculated by the following formulae: Lp = Ln[p/(1 - p)] and V (Lp) = 1/(np) + 1/[n (1 - p)] where Lp = the logit event estimate; Ln = the natural logarithm; p = study level prevalence estimate; V = variance and n = sample size. The pooled logit event estimates were calculated by the DerSimonian and Laird random effects model [25] and back-transformed to prevalence estimates by the following formula: p = e^Lp^/(e^Lp^ + 1): where e = the base of the natural logarithm. The Z test was used to test the significance of the prevalence estimates. The statistical significances of the differences in prevalence estimates between sub-groups were assessed by the Yates corrected Chi Square test [26,27] and the strengths of associations were measured by Odds Ratios (OR). Alpha was set at 0.05.

Microsoft Office Excel 2007 was used to calculate the study level prevalence estimates and standard errors, the logit event estimates and standard errors and to transform the pooled logit estimates to prevalence estimates. The StatCalc epidemiologic calculator of Epi info™ (Version 3.5.1, Center for Disease Control, CDC, USA) was used to compare groups. All other analyses were done by using Stata (Version 11.1, Stata Corp, College Station, Texas).

## Results

### Eligible and excluded studies

**Figure 1:**
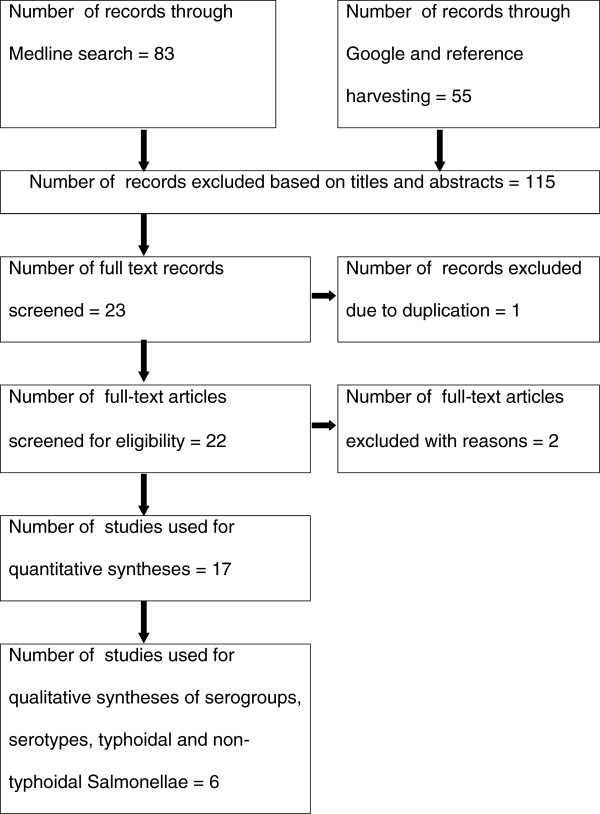
A flow diagram of the selection of eligible studies.

Figure 1 shows the literature search results and the processes in the selection of eligible studies. A total of 138 studies were found of which 115 were excluded based on the titles and abstracts and one was excluded due to duplication. Of the 22 articles screened for eligibility, one study was excluded for it neither provided the number of samples nor separately presented the serogroups isolated from patients and controls [28]. Another study was excluded because it was conducted in a rehabilitation camp [29]. A total of twenty studies were considered eligible for qualitative and quantitative syntheses [30-49]. Seven studies [30,32,35-37,41,46] were used to estimate the prevalence of *Salmonella* in diarrheal patients. Four studies [31,33,42,46] were used to estimate the prevalence of *Salmonella* in febrile patients. Eight studies [34,37-40,43-45] were used to estimate the prevalence of *Salmonella* in apparently healthy subjects. Of the hospital based studies, five reported serogroups [32,35,46-48]; six reported the proportions of non-typhoidal and typhoidal *Salmonella*[32,35,46-49] and two reported serotypes [46,47]. Four non-hospital based studies reported serotypes [34,40,44,45].

### Characteristics of the eligible studies

Table 1 presents the characteristics of the eligible studies. The studies were conducted between 1974 and 2012 in Central, Eastern, Western, Southern and Northern Ethiopia. Except in three studies [47-49], the samples were either stool or blood or both.

**Table 1:** Characteristics of the eligible studies

**First authors**	**Location**	**Year of study**	**Setting**	**Age group**	**Sample source**	**Sample number**	**Positive**	**(%)**
Ashenafi [30]	Addis Ababa	1982-1983	Hospital	Adults	s	1000	45	4.5
Ghiorgis [31]	Addis Ababa	na	Hospital	Children	b	634	28	4.4
Mache [32]	Addis Ababa	1995	Hospital	Adults	s	700	45	6.4
Asrat [33]	Addis Ababa	1996-1998	Hospital	Adults	b	1110	9	0.8
Nyeleti [34]	Addis Ababa	1999	ap	Adults	s	300	18	6
Mache [35]	Jimma	2000	Hospital	Children	s	384	59	15.4
Andualem [36]	Addis Ababa	2000-2001	Hospital	All	s	205	22	10.7
Awole [37]	Jimma	2001	Hospital	Adults	s	152	11	7.2
Awole [37]	Jimma	2001	ho	Adults	s	220	0	0
Birhaneselassie[38]	Dilla	2002	fh	Adults	s	107	1	0.9
Andargie [39]	Gondar	2003	fh	Adults	s	127	0	0
Zewdu [40]	Addis Ababa	2004	sm	Adults	s	68	5	7.4
Reda [41]	Harar	2007	Hospital	Adults	s	244	28	11.5
Abera [42]	Bahir Dar	2009	fh	Adults	s	384	6	1.6
Zenebe [43]	Jimma	2009-2010	Hospital	Adults	b	260	1	0.4
Addis [44]	Addis Ababa	2010	df	Adults	s	22	3	13.6
Dagnew [45]	Gondar	2012	fh	Adults	s	300	4	1.3
Beyene [46]^a^	Addis-Jimma	2006	Hospital	Children	s/r	1003	48	4.8
Beyene [46]^a^	Addis-Jimma	2006	Hospital	Children	b	222	17	7.7
Beyene [46]^a^	Addis Ababa	2004-2005	Hospital	Children	na	na	48	na
Gebre-yohannes [47]	Addis Ababa	1974-1981	Hospital	All	m	na	216	na
Gedebou [48]	Addis Ababa	1975-1980	Hospital	All	m	na	165	na
Wolday [49]	Addis Ababa	1993-1996	Hospital	na	m	na	110	na

Addis-Jimma = Addis Ababa and Jimma, ap = abattoir personnel, b = blood, df = dairy farm attendants, fh = food handlers, ho = samples taken in a hospital from patients with complaints other than gastro-enteritis and fever, m = mixed samples, na = not available, s = stool, sm = supermarket personnel, s/r = stool or rectal swabs.

^a^The number of stool and blood samples and *Salmonella* positive samples were extracted from the Thesis but other data were extracted from the article.

Data from 5898 patients and 1528 apparently healthy subjects were considered for quantitative syntheses. The prevalence of *Salmonella* in stool samples of patients and apparently healthy subjects ranged from 4.5% to 15.4% and from 0% to 13.6% respectively. Of the 707 Salmonellae isolated from specimens taken from patients, 597 isolates were serogrouped and 329 were serotyped. Of the 37 Salmonellae isolated from apparently healthy subjects, 33 isolates were serotyped.

### Risks of bias

Table 2 presents the microbiological methods employed in the isolation and identification of *Salmonella*. Pre enrichment was reported in four studies. Two studies reported two selective enrichment media, and six studies reported two plating media. Enrichment with Selenite containing media, plating with Salmonella Shigella agar and Desoxycholate Citrate agar were reported in 12, 7 and 5 of the studies respectively. Only one study reported pre enrichment broth, two selective enrichment and three plating media. Biochemical tests were reported in all studies but one and slide agglutination tests were reported in 10 studies.

**Table 2:** **Microbiological methods used to isolate and identify *Salmonella***

**First author**	**Pre-enrichment**	**Selective and differential media**	**BT**	**SAT**
Ashenafi [30]	nr	SB, MA, BGA	yes	Yes
Mache [32]	nr	SF, MA, BGA,SSA	yes	Yes
Mache [35]	nr	SB, MA, SSA	yes	Yes
Andualem [36]	nr	SF, MA, SSA	yes	nr
Awole [37]	nr	MA, DCA, SSA	Yes	nr
Beyene [46]	nr	SF, DCA, XLD	yes	Yes
Reda [41]	nr	SF, DCA, XLD	yes	nr
Asrat [33]	nr	SB, MA, BGA	yes	Yes
Zenebe [43]	BHI	BA, MA	yes	Yes
Beyene [46]	BHI	DCA, XLD	yes	Yes
Nyeleti [34].	nr	RV, MA , BPLS	yes	Yes
Awole [37]	nr	MA, DCA, SSA	Yes	nr
Berhane selassie [38]	nr	SB, DCA	yes	Yes
Andargie [39]	nr	MA, BA, SSA	nr	nr
Zewdu [40]	BPW	SC, RV, BPLS, XLD, RA	yes	Yes
Abera [42]	nr	SF, SSA	nr	nr
Addis [44]	BPW	SC, RV, XLD	yes	nr
Dagnew [45]	nr	SF, SSA	yes	nr

BA = Blood agar, BHI = Brain Heart infusion agar, BGA = Brilliant Green agar, BPLS = Brilliant Phenol Lactose –Sucrose agar, BPW = Buffered Peptone Water, BT = Biochemical test, DCA = Desoxycholate Citrate agar, MA = MacConkey agar, nr = not reported, RA = Rambach agar, RV = Rappaport Vassilliadis, SAT = Slide Agglutination Test; SSA = *Salmonella* Shigella agar, SB = Selenite broth, SC = Selenite Cystine broth, SF = Selenite-F broth, XLD = Xylose Lysine Desoxycholate agar.

Outliers were detected in the funnel plots of the prevalence estimates of *Salmonellae* isolated from stool samples of diarrheal patients and apparently normal individuals (plots not shown). The pooled prevalence of *Salmonella* in diarrheal patients was not changed by the Duval and Tweedie method and the lowest and highest single study omitted estimates were 6.75% and 9.07% respectively. The pooled prevalence of *Salmonella* in apparently healthy individuals was slightly higher than the estimate calculated by the Duval and Tweedie method and the lowest and highest single study omitted estimates were 0.58% and 1.27% respectively. All single study omitted pooled estimates were within the 95% confidence intervals of the respective overall pooled estimates in diarrheal patients and apparently normal individuals.

### Forest plots

**Figure 2:**
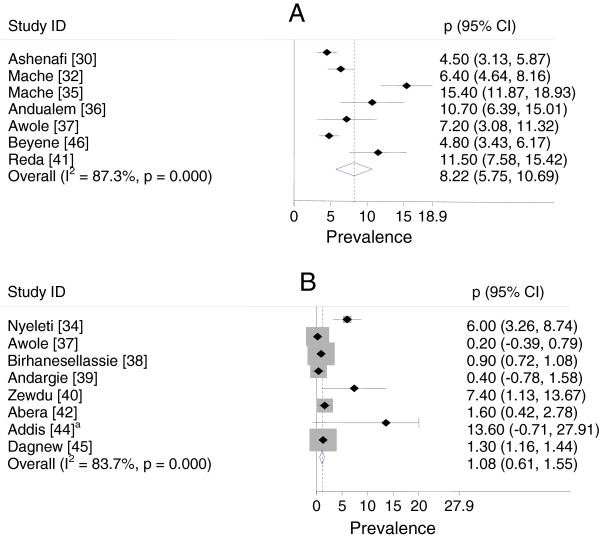
**Forest plot of the prevalence of *Salmonella *isolated from stool samples of patients (A) and apparently healthy subjects (B).**^a^The upper confidence limit of Addis [44] is off the scale and truncated.

Figure 2 presents forest plots of the untransformed prevalence estimates of *Salmonella* isolated from stool samples of diarrheal patients and apparently healthy subjects. The percentage of the variations in prevalence estimates attributable to heterogeneity (I^2^) were 87.3% in diarrheal patients and 83.7% in apparently normal subjects. All study level prevalence estimates of *Salmonella* in diarrheal patients were significantly different from zero (p < 0.05). Three study level prevalence estimates of *Salmonella* in apparently healthy subjects were not significantly different from zero (p > 0.05).

### Pooled prevalence estimates

The pooled estimates calculated based on the untransformed and logit transformed estimates are presented in Table 3. The differences in the pooled estimates of the untransformed and transformed data were negligible. The prevalence of *Salmonella* was higher in diarrheal children than in diarrheal adults (X^2^ = 10.93; p = 0.000; OR = 1.59 (95% CI = 1.20, 2.10), in febrile children than in febrile adults (X^2^ = 48.27; p = 0.000; OR = 8.16 (95% CI = 3.97, 17.25) and in individuals exposed to animal products or animals (super market personnel, dairy farm attendants and abattoir personnel) than in food handlers (X^2^ = 27.93; p = 0.000; OR = 5.62 (95% CI = 2.70, 11.88).

**Table 3:** **Pooled prevalence estimates of *Salmonella *** **in patients that sought medical attention and in carriers**

**Group**	**Untransformed data**		**Logit transformed data**^**c**^	
	**Prevalence (95% CI)**	**I**^**2**^**, p value**^**d**^	**Prevalence (95%CI )**	**I**^**2**^**, p value**^**d**^
Diarrheal patients^a^	8.22 (5.75, 10.69)	87.3, 0.000	7.93 (5.30, 11.70)	90.8, 0.000
Diarrheal adults	5.58 (3.99, 7.17)	44.6, 0.164	5.68 (4.26, 7.53)	49.3, 0.139
Diarrheal children	9.97(-0.41, 20.36)	96.7, 0.000	8.72 (2.64, 25.18)	97.5, 0.000
Febrile adults	0.66 (0.19, 1.13)	0.0, 0.424	0.76 (0.41, 1.40)	0.0, 0.516
Febrile children	5.66 (2.52, 8.80)	64.4, 0.094	5.71 (3.31, 9.68)	70.5, 0.066
Over all carriers	1.08 (0.61, 1.55)	83.7, 0.000	2.75 (1.23, 6.17)	76.1, 0.000
Food handlers	1.09(0.73, 1.44)	79.2, 0.002	1.33 (0.75, 2.36)	0.0, 0.793
Others^b^	6.45 (3.97, 8.92)	0.0, 0.562	6.83 (4.69, 9.85)	0.0, 0.391

^a^Diarrheal adults and children.

^b^Individuals in contact with animal products or animals.

^c^The pooled prevalence estimates of the logit transformed data were the summary measures.

^d^The p values are estimated by the Cochran’s Q test.

### Serogroups and serotypes

Typhoidal *Salmonella* (*S*. Typhi) accounted for 42.1% of the total isolates (Table 4) and for 8.7% of the isolates of children origin. Six serogroups were identified from patients (Table 5). Serogroup D occurred more frequently than serogroups C and B. In children, serogroup C ranked first followed by serogroup D. Thirty serovars were identified from specimens taken from patients of which *S*. Concord, *S*. Typhi, *S*. Typhimurium and S. Paratyphi were the first four dominant serotypes that accounted for 82.1% of the serotyped isolates (Table 6). In non hospital based studies, six serovars were identified: *S*. Anatum (7/33), *S*. Dublin (4/33), *S*. Meleagridis (5/33), *S*. Newport (5/33), *S*. Rough form (2/33) and *S*. Typhi (10/33).

**Table 4:** **Frequencies and percentages of *S.*** **Typhi isolated from samples taken from patients**

**Study year**	**Number of isolates**	**Number of *S.*** **Typhi**	**%**	**Authors**
1975-1980	164	123	75	48
1974-1981	216	105	48.6	47
1993-1996	110	48	43.6	49
1995	45	7	15.6	32
2000	59	13	22	35
2004-2006	113	2	1.8	46
1974 -2006	707	298	42.1	

**Table 5:** Frequencies (%) of serogroups identified from samples taken from patients

**Serogroup**	**Number and percentage of serogroups**					
	[48]	[47]	[32]	[35]	[46]	**Total (%)**
	**No. (%)**	**No. (%)**	**No. (%)**	**No. (%)**	**No. (%)**	**No. (%)**
A	7 (4.3)	4 (1.9)	4 (8.9)	5 (8.5)	0	20 (3.4)
B	16 (9.8)	44 (20.4)	11 (24.4)	17 (28.8)	13 (11.5)	101(16.9)
C	17 (10.4)	42 (19.4)	14 (31.1)	13 (22)	86 (76.1)	171(28.6)
D	123 (75)	120 (55.6)	13 (28.9)	21(35.6)	9 (8)	286 (47.9)
E	1(0.6)	6 (2.8)	3 (6.7)	3 (5.1)	2 (1.8)	15 (2.5)
M	0	0	0	0	3 (2.7)	3 (0.5)
Total	164	216	45	59	113	597

**Table 6:** Frequencies (%) of serotypes isolated from samples taken from patients

**Serotypes**	**Number of isolates**		
	[47]	[46]	**Total (%)**
*S.* Concord	27 (12.5)	85 (75.2)	112 (34 )
*S.* Typhi	105 (48.6)	2 (1.8)	107 (32.5)
*S.* Typhimurium	24 (11.1)	7 (6.2)	31 (9.4)
*S.* Paratyphi	18 (8.3)	2 (1.8)	20(6.1)
Others	42 (19.4)	17 (15.0)	59(17.9)
Total	216	113	329 (100)

## Discussion

Studies on human Salmonellosis in Ethiopia began in the 1970s. However, the number of studies so far carried out is small and do not include all segments of the population and geographic regions of the country. Most studies were carried out in Addis Ababa and risk factors that potentially influence the occurrence of the disease including the diversity of serovars were not sufficiently addressed to provide a comprehensive picture of the epidemiology of the disease. In addition to the small number of studies, the aggregate reporting and lack of distinct data by sample type and age group were constraints in estimating the prevalence of serogroups and serotypes.

The microbiological methods employed in the studies might have underestimated the numbers of *Salmonella* positive samples. The use of pre enrichment broth increases culture sensitivity and the numbers of isolates could differ by types of media [50]. *Salmonella* strains have different characteristics in enrichment media and there could be a differential recovery of one strain over another [51]. As the rates of isolation of strains differ in different media versions, multiple enrichment protocols were suggested to ensure the isolation of different strains [52]. The recovery rate of *Salmonella*, the sensitivities and positive predictive values of plating media were also reported to be higher after enrichment than direct culture [53].

The substantial variations in prevalence estimates attributable to heterogeneity could be due to differences in the study populations and the microbiological methods used to detect *Salmonella*. However, the pooled estimates were not sensitive to the effects of outliers and depict the importance of the disease as the random effects model considers the studies as samples of all potential studies.

The pooled prevalence of *Salmonella* in diarrheal patients is comparable with the estimate for SSA (8.36%) [54]. The present estimate appears to be lower than its actual occurrence due to the apparently lower sensitivities of the microbiological methods used in most of the studies. In addition, several patients do not seek medical attention for less severe gastroenteritis and are unable to cover medical costs for even severe cases. However, the widespread occurrences of several factors including malnutrition and under nutrition, HIV-AIDS, the close relationship between man and animals, the widespread backyard slaughtering practices, the raw meat consumption habits, the unhygienic food handling practices and the water sources of the larger segment of the population are suggestive evidences of its higher occurrence than is estimated in this study.

The higher occurrence of Salmonellosis in children compared to adults suggests a higher vulnerability of children to *Salmonella* infections. Disseminated diseases were reported in children with severe protein-energy malnutrition [55]. The bacterium was isolated from stool samples of pediatric patients [56] and demonstrated in malarial blood smears of febrile children [57]. Regardless of the small number of studies considered in this study, the pooled estimates show the importance of the disease in Ethiopian children as is the case in other countries in SSA [58].

Salmonellae were isolated from individuals working in food catering establishments, super market workers, dairy farm attendants and abattoir personnel. Although these individuals could either be asymptomatic or precocious or acute or convalescent or chronic carriers, all are potential sources of outbreaks. In the USA, a few food service employees were implicated for an outbreak that caused 617 (6.3%) illnesses [59].

The proportion of *S*. Typhi isolates tends to decline as of the 1970s while NTS infections show a relative increase. As the data were drawn from urban areas, improvements in hygienic and sanitary measures practiced in the years after the 1970s might have reduced the occurrence of typhoidal infections in urban areas. However, the proportional decreases in typhoidal infections do not reflect the substantial improvements in the hygienic and sanitary practices of the general population.

Serogroups D, C and B were the major serogroups and their occurrences differ by age group. Whilst serogroup C ranked first in children [35,46], serogroup D was dominant in samples predominantly taken from adults [47]. Higher indexes of invasion were also recorded for serogroup D [48] and serogroup C [46]. In addition to the differences by age group, the proportions of serogroups may differ by sample type and study years.

*S*. Concord and *S*. Typhimurium were the first and third ranking NTS isolated from patients. In a global analysis of reports from 26 countries, *S*. Enteritidis (65%) and *S*. Typhimurium (12%) were the dominant serotypes isolated from 2000 to 2002 [6]. In Ethiopia, *S*. Enteritidis accounted for 1.8% of the isolates [46,47] and less than five isolates were isolated from samples of cattle [60], sheep [61], pigs [62,63] and chicken [64]. The differences in the preponderance of serovars across countries could be due to the variations in the epidemiology of the serotypes that may involve animal as well as environmental factors.

*S*. Concord was the first ranking serovar isolated from patients. Its occurrence was higher in samples taken from children [46] than in samples predominantly from adults [47]. The bacterium was isolated from Ethiopian children adopted in several European countries and the USA [65-69] but not demonstrated in samples taken from domestic food animals and food items of animal origin. As *S*. Concord was reported to be highly invasive [46] and multi drug resistant [46,47,65-69], it could be a serious cause of morbidity and mortality in children.

*S.* Typhi and *S*. Paratyphi are causes of enteric fever with similar disease patterns [69]. In the 1980s, typhoid fever was diagnosed in 1.1% of the Ethiopian immigrants in Israel [70] and a case fatality rate of 15.7% was reported in hospital admitted Ethiopian children [71]. In a recent study on patients with febrile illnesses, typhoid fever was recorded in 5.85% of the patients with a higher occurrence in children aged three to 14 years (6.6%) compared to children aged 15 to 17 years (1.1%) [72]. Although a systematic epidemiological study is not available, the lower living standard of the general population is a suggestive evidence that enteric fever is a threat in present day Ethiopia.

*S*. Typhimurium was the third ranking serovar that accounted for 15.3% of the NTS isolates [46,47] and its occurrence is within the range of its global preponderance (10-30%) [73].This serotype was isolated from slaughtered cattle, sheep and camels [56-58,74-76], minced beef [38,64] and chicken samples [40,77,78] in Ethiopia and reported to be a common cause of invasive disease [4,12] with a higher mortality rate in AIDS patients [79].

Of the isolates recovered from stool samples of carriers, *S*. Dublin and *S.* Newport could be of significant public health concerns. *S.* Newport was a cause of an outbreak in Gondar College of Health Sciences, Ethiopia where 79 (23%) students had manifest disease [80] and it was isolated in meat samples collected from supermarkets [40] and in samples taken from slaughtered animals [63,74,75]. *S*. Dublin was isolated from human clinical samples [47], slaughtered cattle [34,60] and from meats collected from markets [34,40,64]. Significant associations of *S*. Dublin infection with raw milk consumption habits, higher invasion (65%), hospital admission (78.8%) and mortality rates (19.5%) were reported [81].

### Limitations and implications of the study

The patient data were collected from Hospitals that provided services to both urban and rural inhabitants but the origin of the study subjects were not reported. However, it appears that most patients could be from urban areas because of the relatively better accesses to health care settings. Therefore, the pooled estimates are more applicable to patients in urban areas than to patients in rural areas.

The results of this study have several implications in clinical practices, policy issues and intervention measures and in research undertakings. First, as drug sensitivity tests are not routinely carried out in several clinical settings of the country, antimicrobials known to be effective against the dominant serovars could be used as empirical therapeutic agents. Secondly, the considerable occurrences of *Salmonella* in patients and apparently healthy subjects imply the need for a policy and intervention measures to promote public hygiene and regularly screen individuals that are in contact with food items meant for public consumption. In addition, the limited number of studies and the occurrence of serotypes of significant national and international concerns [46,62,63,65-68], entail the need for a large scale study to describe the epidemiology of the disease at a national level. The pooled estimates are more reliable than the single study estimates and could be used in the design of further studies.

## Conclusion

The prevalence of Salmonellosis is considerable and a few serotypes that included *S*. Concord, *S*. Typhi, *S*. Typhimurium, and *S*. Paratyphi are the major causes of infections. There is a need of a policy and intervention measures to promote public hygiene and regularly screen individuals in contact with food items for public consumption. Further studies are required to adequately describe the epidemiology of human Salmonellosis in Ethiopia.

## Competing interests

The author declares no competing interests.

## Author’s contributions

GT conceived the study design, searched the literature, extracted the data, analyzed the data, interpreted the results and drafted the manuscript.

## Pre-publication history

The pre-publication history for this paper can be accessed here:

http://www.biomedcentral.com/1471-2334/14/88/prepub

## Supplementary Material

Additional file 1 PRISMA Checklist. Click here for file
